# Enhancement in Mechanical Properties of Glass/Epoxy Composites by a Hybrid Combination of Multi-Walled Carbon Nanotubes and Graphene Nanoparticles

**DOI:** 10.3390/polym15051189

**Published:** 2023-02-27

**Authors:** Seshaiah Turaka, Aswani Kumar Bandaru

**Affiliations:** 1 Department of Mechanical Engineering, QIS College of Engineering and Technology, Ongole 523002, India; 2 Bernal Institute, School of Engineering, University of Limerick, V94 T9PX Limerick, Ireland

**Keywords:** glass, epoxy, carbon nanotubes, graphene, flexural, interlaminar shear

## Abstract

In this work, an attempt was made to improve the mechanical performance of glass fibre-reinforced polymer composites by adding multi-walled carbon nanotubes (MWCNT) and graphene nanoparticles (GNP) and their hybrid combination at different weight fractions (0.1 to 0.3%). Composite laminates with three different configurations (unidirectional [0°]_12_, cross-ply [0°/90°]_3s_, and angle-ply [±45°]_3s_) were manufactured using the compression moulding method. Characterisation tests such as quasistatic compression, flexural, and interlaminar shear strength properties were carried out per ASTM standards. Failure analysis was carried out through optical and scanning electron microscopy (SEM). The experimental results showed a substantial enhancement with the 0.2% hybrid combination of MWCNTs, and GNPs showed 80% and 74% in the compressive strength and compressive modulus, respectively. Similarly, flexural strength, modulus, and interlaminar shear strength (ILSS) increased by 62%, 205%, and 298%, respectively, compared to neat glass/epoxy resin composite. Beyond the 0.2% of fillers, the properties started to degrade due to the agglomeration of MWCNTs/GNPs. The order of layups per mechanical performance was UD, followed by CP and AP.

## 1. Introduction

Fibre-reinforced polymer matrix composites have been widely used in applications such as structural, aerospace, naval, wind, high-pressure pipes, etc. Glass fibre is one of the extensively used reinforcements due to its low cost, lightweight, high strength under static/dynamic loads, and because it is less corrosive [1]. For high-strength applications, the mechanical characteristics of the conventional laminated structures can be increased by adding graphene (GNP) [2,3,4,5] and inorganic or organic fillers to the epoxy resin [6,7]. Numerous studies revealed that adding nano- and micro-scale particles enhance the properties and effectiveness of composites [8,9,10,11,12,13]. Several methods have used filler materials such as nanoclay, nanorods, nanodiamonds, single/multi-walled carbon nanotubes (MWCNT) etc., to alter the properties of composite materials [14,15,16,17,18,19,20,21,22]. The uniform distribution of nanoclay into the epoxy resin increases the fatigue strength and modulus of elasticity while slightly decreasing the tensile strength with the increase in nanoclay beyond a certain percentage [23,24]. Dispersing small quantities of carbon nanotubes and nanodiamonds in neat polyester resin increases the mechanical properties of the composites [25,26,27]. Prabhakaran et al. [28] also reported improvements in mechanical properties such as compression, shear, bending, and toughness of unstitched plain weave/polyester resin with carbon and biaxial non-crimp/polyester composites over neat polyester resin-based composites [28]. Adding 3–8% nanoparticles, i.e., MWCNT/carbon nanofillers (CNF), into the neat epoxy matrix enhanced the mechanical properties. It was found that 5% of MWCNT/CNF increased the fracture toughness, flexural strength, and electrical conductivity by 18%, 28%, and 22%, respectively, in hybrid nanocomposites [29].

The manufacturing processes used to fabricate the laminated composite and the additions of nanofillers influence the mechanical properties. Guo et al. [30] reported that using a mechanical shear mixer followed by a sonicator with in situ polymerisation for carbon/vinyl ester composites with nanoclay improved the mechanical properties and interface. Mechanical properties can also be enhanced through the uniform distribution of nanoparticles in the virgin polymeric epoxy matrix using acoustic ultrasonication [31]. Infusion of AR10 and AR50 CNFs improved the specific strength, specific modulus, and flexural strength of two-phase and three-phase carbon fibre/epoxy composites [32]. The dispersion of Al_2_O_3_ nanoparticles within the epoxy also showed little influence on the mechanical properties [33]. The single-walled carbon nanotubes, double-walled carbon nanotubes, and MWCNTs with epoxy composites showed significant improvement in mechanical properties with 0.5% fillers [34]. The influence of hybrid kenaf-aramid/epoxy composite with CNFs for defence applications exhibited increased tensile strength and impact loads [35]. The strength, stiffness, and toughness of particulate nanocomposites can be enhanced by the appropriate dispersion of CNTs through acoustic cavitation [36]. The intertwined E-glass/polyester nanocomposites with 0.1–0.4% CNFs significantly increased the flexural and elastic modulus compared to the conventional E-glass/polyester composite [37]. The aluminium alloy reinforced with particulate nano Al_2_O_3_ revealed significant development in mechanical properties [38]. Dispersion of micro glass fibres at 0.1, 0.2, and 0.3% to the geopolymer composites improved the compressive strength, Young’s modulus, fracture toughness, and rigidity [39]. Using a high-speed mechanical mixer to disperse polypropylene to glass fabric improved the flexural and impact strength [40], Chatterjee et al. [41] reported that the hybrid combination of GNP and MWCNT improved fracture toughness and the flexural modulus by 82% and 9% for 2% and 1% of GNPs. Recently, Zhang et al. [42] reported that the hybrid combination of MWCNT and GNPs enhanced the quasi-static fracture toughness and dynamic compressive strength by 75% and 82%, respectively. However, these properties were improved when 0.5% fillers were added. Beyond this percentage, the properties started decreasing. In addition to the mechanical properties, other properties such as physiochemical, heat release rate, and electrical properties were improved by CNT, MWCNT and GNP [43,44,45,46,47]. There are other authors [48,49] who studied the bending and in-plane compressive properties of self-sensing sandwich structures using graphene-coated glass fibre for aerostructures [48]. The reduced graphene oxide (rGO) coated on glass fabric enhanced the dominant loading modes experienced by composite aerostructures at various load rates. Table 1 presents the summary of various nanofillers and corresponding improvements in properties.

The above-mentioned literature reported that the composite’s performance could be improved with micro and nanofillers at specific weight fractions. Most of the studies concentrated on using either MWCNT, GNP, or nanoclay. These studies proved that adding these nanofillers improves composites’ mechanical performance. However, very few studies reported the hybridisation of nanofillers with a combination of MWCNT and GNP [41,42,47]. These studies were limited to static fracture toughness, dynamic compressive strength, and electrical properties. Therefore, this study presents a compressive report on the GFRP composites filled with MWCNT, GNP, and a hybrid combination of MWCNT + GNP at different weight percentages. Composite laminates with different layups, such as unidirectional (UD), cross-ply (CP), and angle ply (AP), were manufactured. During manufacturing, MWCNT and GNP were added to the epoxy resin at different wt%, explained in Section 2. The quasistatic compression, flexural, and interlaminar shear strength (ILSS) tests were performed. Failure analysis was performed using optical and scanning electron microscopy (SEM) (Section 3).

## 2. Materials and Methods

### 2.1. Materials

Unidirectional glass fibres (200 gsm), multi-walled carbon nanotubes (MWCNTs) and graphene nanoparticles (GNPs) with a 5–10 nm average diameter were obtained from Arun Fabrics Pvt. Ltd. and United Nano Tech Innovations Pvt. Ltd., Bangalore, India, respectively. The epoxy resin (LY-556) and styrene (hardener) were supplied by Tirven Industries Pvt. Ltd., Hyderabad, India. The physical and mechanical properties of the resin and nanofillers are shown in Table 2.

### 2.2. Manufacturing of Nanocomposites

Araldite (LY-556) 55%, hardener (HY-951) 49%, and accelerator (DY-080) 0.28% were used to prepare epoxy resin. MWCNT/GNP fillers were used in different weight percentages varying from 0.1 to 0.3. First, MWCNT, GNP, and MWCNT + GNP were dispersed as per the above-mentioned weight percentages. The suspension of epoxy and nanofillers was mixed mechanically using a three roll mill (EXAKT 80E Technologies Germany), which introduced very high shear forces (up to 200,000/s) throughout the suspension. After the dispersion, the hardener and accelerator were added to a vacuum dissolver to avoid trapped air in the suspension. Then, the mixture was placed in a vacuum chamber for 20 min to eliminate the bubbles introduced during the rolling process. The same process was repeated for all the weight percentages.

**Figure 1:**
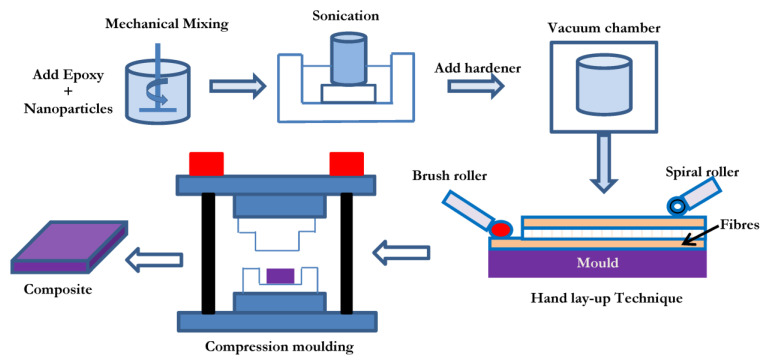
Laminated nanocomposite preparation process cycle.

**Figure 2:**
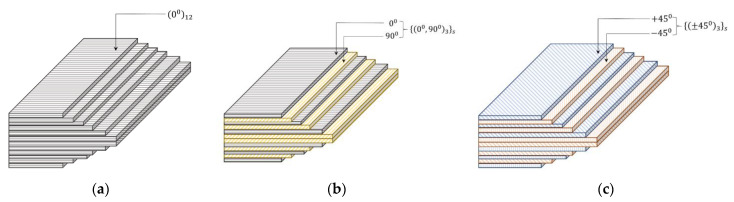
Illustration of laminate stacking sequence: (**a**) UD, (**b**) CP, and (**c**) AP.

Both conventional (MWCNT, GNP) and hybrid (MWCNT + GNP) composites were manufactured by the compression moulding process. A schematic of the manufacturing process is shown in Figure 1. The vacuum of one atmosphere was maintained for about 12–15 h until the end of the curing process at room temperature to constrain the void formation through polymerisation. A mechanical convection oven was used for post-curing at 110 °C for 3 h. Three different configurations of fibres were used: unidirectional (UD), cross-ply (CP), and angle-ply (AP). The stacking sequence of these laminates is shown in Figure 2.

### 2.3. Mechanical Characterisation

The influence of nanofillers with the homogenous and hybrid combination on the mechanical performance of GFRP composites was assessed through the quasistatic compressive, flexural, and interlaminar shear (ILSS) tests. Five samples were tested, and their average values were presented.

#### 2.3.1. Quasistatic Compression

**Figure 3:**
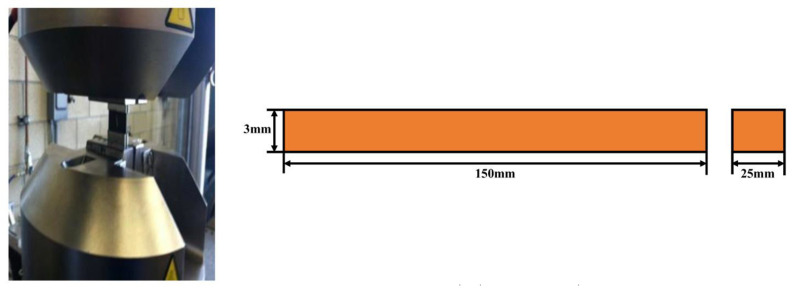
Quasi-static compression test arrangement.

Quasistatic compression tests through the thickness direction were performed as per ASTM D3410 using Instron 785, a universal testing machine (UTM), controlled by a hydraulic servo motor. The sample was tested with a 1.27 mm/min displacement rate, as shown in Figure 3. All samples were sanded and polished, as per the standards to uphold uniformly distributed compressive loading.

#### 2.3.2. Flexural Test

**Figure 4:**
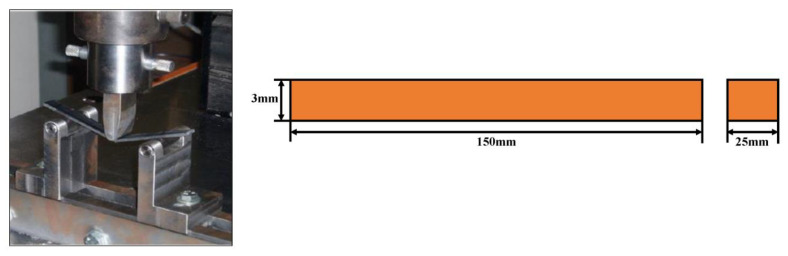
Flexural test arrangement.

Three-point bend tests were performed to analyse the flexural behaviour of the laminates, as per ASTM D7264. The test was performed on an Instron 785 UTM with a 20 kN load cell, with a displacement rate of 2.0 mm/min under room temperature, as shown in Figure 4. From the slope of the stress–strain curve, the flexural modulus was calculated. The specimens were prepared to maintain a span-to-thickness ratio of 32:1. The flexural stress, strain, and flexural modulus were calculated using the equations given in ASTM D7264.

#### 2.3.3. ILSS Test

**Figure 5:**
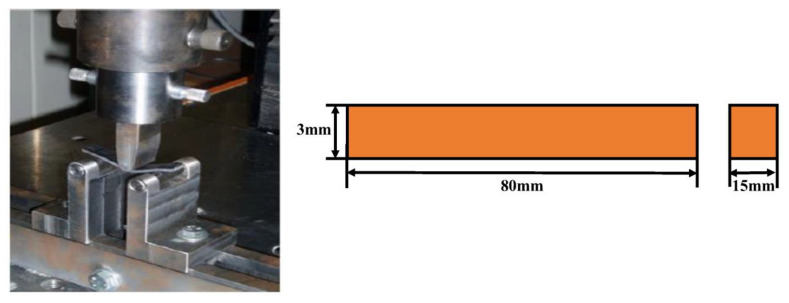
ILSS test arrangement.

The ILSS test was performed as per the ASTM D2344 standard, as shown in Figure 5. The specimen was placed on a horizontal shear test fixture and applied transverse loading was at a rate of 1.27 mm/min until the failure of the specimen. The tests were performed on an Instron 785 UTM with a 20 kN load cell. The upper roller diameter was 10 mm and the diameter of the lower rollers was 5 mm.

#### 2.3.4. Failure Analysis

Optical microscopy was used to identify the failure of the samples at the macro scale. The SEM micrographs of failed laminates were investigated through SEM using a JEOL JSM 5800. For easier understanding, the morphology of fractured samples was studied using higher-resolution SEM micrographs. The samples are as per the standards and coated accordingly.

## 3. Results and Discussion

The following sections presented the quasistatic compression, flexural, and ILSS properties of GE composites filled with different MWCNT, GNP, and hybrid combinations of MWCNT + GNP. The influence of nanofillers on the GE composites with different fibre orientations such as UD, CP, and AP was presented. In the case of failure analysis, due to a huge number of figures, **only the failure of UD laminates was presented** in the main manuscript. The SEM failure of **CP and AP laminates was provided as a supplementary file**.

### 3.1. Dispersion of Nanofillers

**Figure 6:**
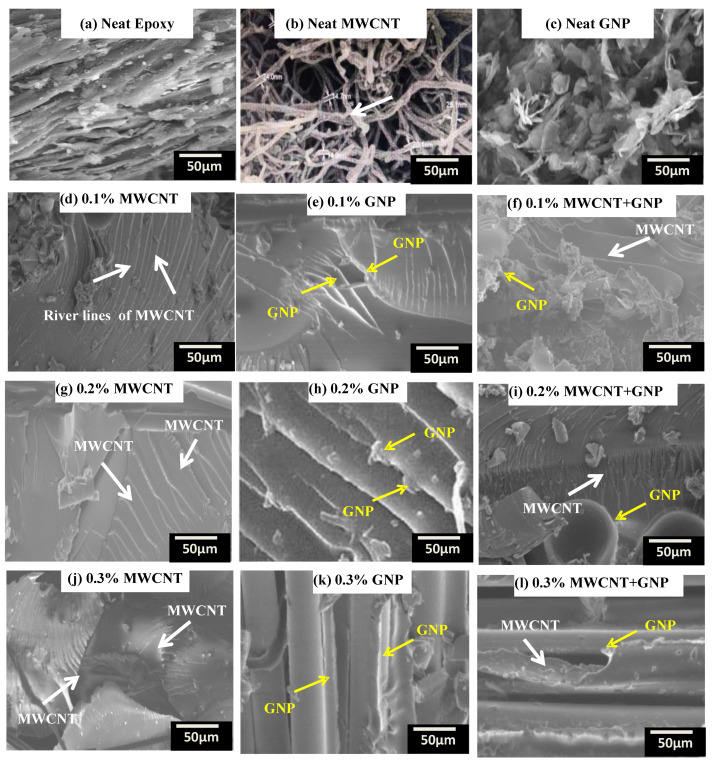
Dispersion of nanofillers in epoxy at different weight percentages.

The dispersion of nanofillers in the matrix is shown in Figure 6 through SEM micrographs. Micrographs in Figure 6g–i with 0.2% MWCNT, 0.2% GNP, and 0.2% (MWCNT + GNP) dispersion showed good and uniform dispersion with no noticeable agglomeration. The MWCNT and GNP fillers interlocked and entangled with the polymer chains of the resin; some MWCNT and GNP were broken in a brittle manner and pulled out at the ends of the surface. The 0.3% MWCNT and GNP fillers showed agglomeration in the solution, which caused the van der Waals interactions. Agglomeration of MWCNT and GNP reduces the strength of the nanocomposites by the stress concentration effect. To overcome the attraction of van der Waals forces, many researchers suggested maintaining proper ultrasonicator and high-speed shear mixing [52]. However, optimum loading and uniform dispersion of nanofillers are key input parameters to promote interfacial bonding between CNF matrix interfaces to carry the efficient load between the two constituents of nanofillers [36].

### 3.2. Quasistatic Compression Properties

**Figure 7:**
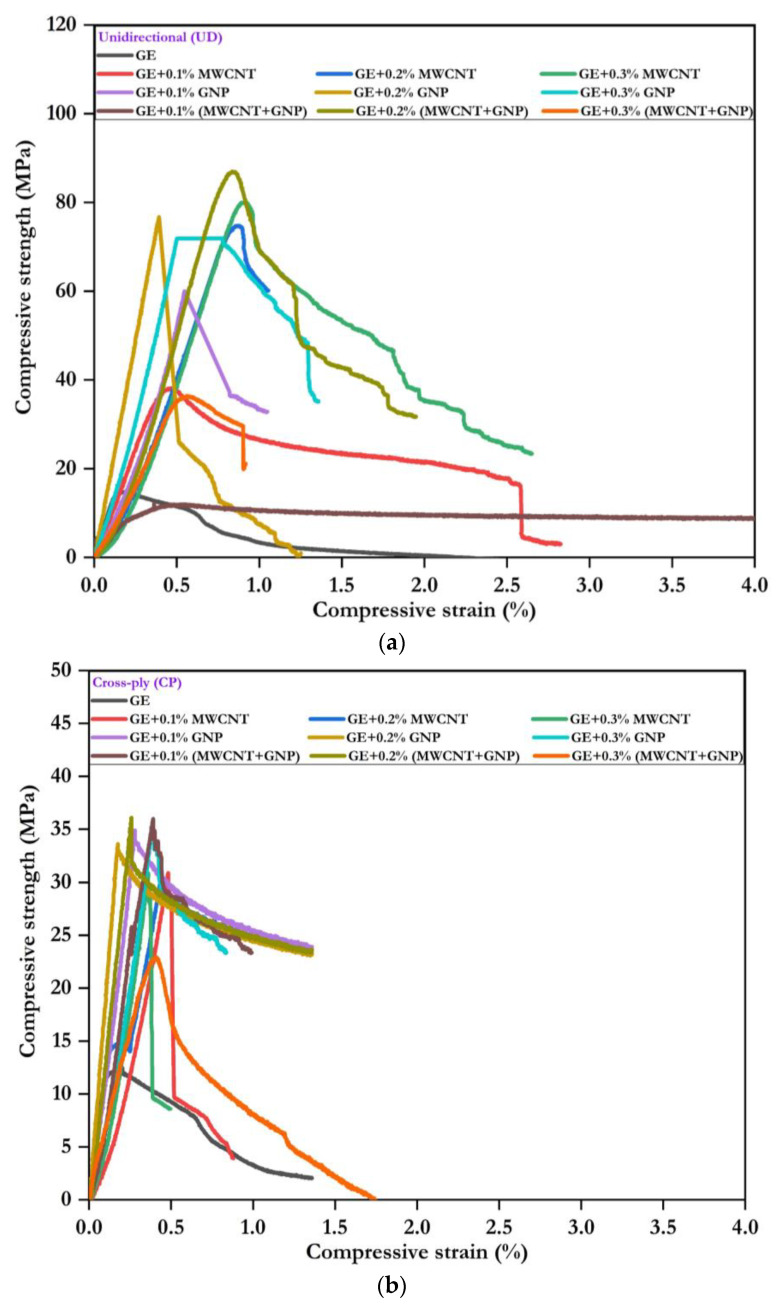

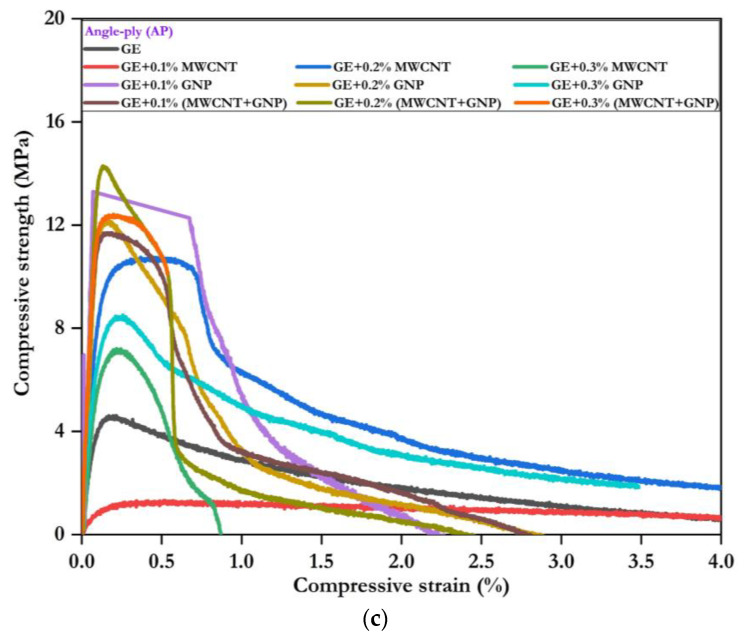
Compressive stress–strain response: (**a**) UD, (**b**) CP, and (**c**) AP.

The load deflection results data were logged and converted to the stress–strain plot by dividing the load by the original cross-sectional area of the specimen and deflection by the thickness of the specimen. The modulus was calculated from the proportional limit zone slope of the stress–strain curve. The quasistatic compression stress–strain response of UD, CP, and AP laminates with different weight fractions of nanofillers are shown in Figure 7.

From the compressive stress–strain response (Figure 7a–c), it was found that the incorporation of fillers in hybrid form (MWCNT + GNP) enhanced the strength and modulus of the UD laminate. Depending on the layup of the composite, the response was different for different composites. After reaching the maximum stress, the samples failed in all the cases in all the composites. Brittle failure was observed in each type of laminate, and no obvious yield point was found. There was a slight improvement in the strain at the peak stress due to the addition of hybrid nanofillers. The 0.2% (MWCNT + GNP) laminate showed the best compressive stress and modulus improvement. The combination of MWCNT + GNP provided resistance against crack propagation. The addition of nanofillers reduced the voids and improved the strength and modulus of the laminates. However, the percentage of nanofillers added plays a role in deciding the interfacial strength between the matrix and fibre. From 0.1% to 0.2% addition of the nanofillers, the response was improved with the load. However, beyond 0.2% of the nanofillers, i.e., at 0.3%, the response was decreased due to possible agglomeration of filler materials.

**Figure 8:**
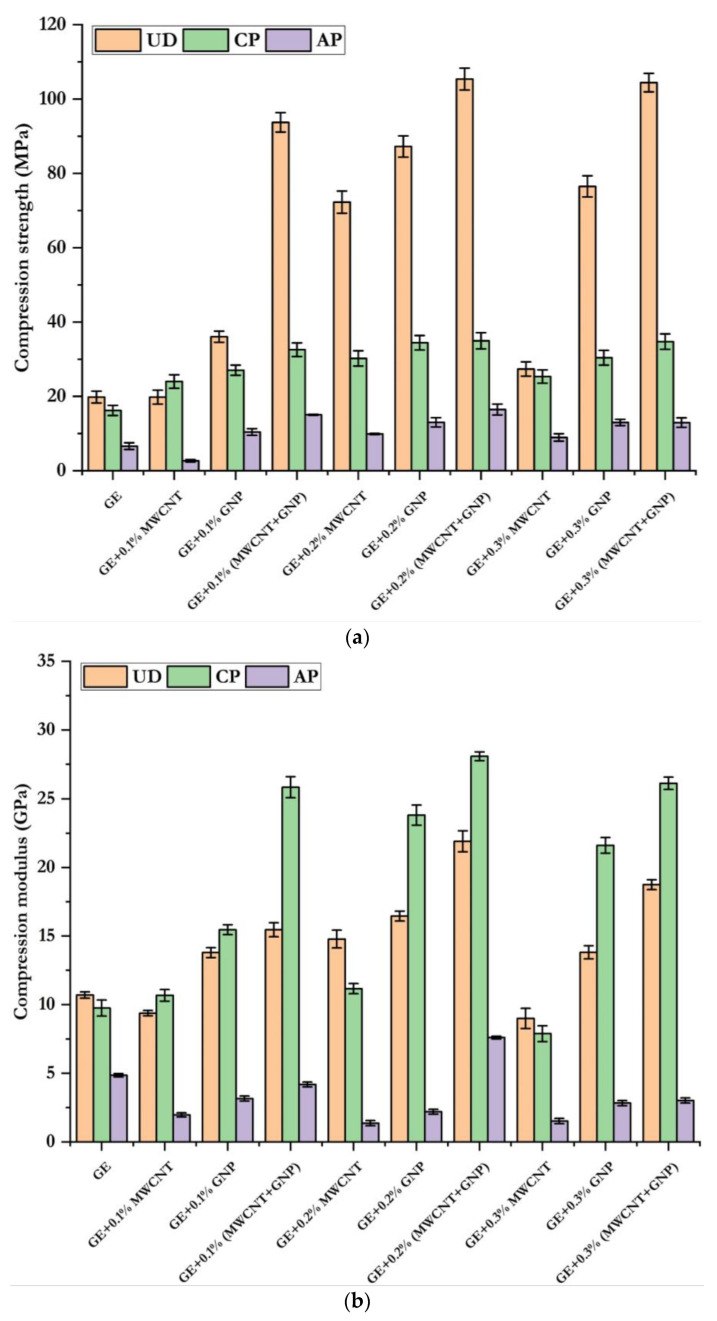
Compressive properties of GE composites: (**a**) compressive strength (**b**) compressive modulus.

Figure 8 compares GE’s compressive strength, modulus, and nanocomposites with different layups. From the comparison, it was understandable that the addition of nanofillers significantly enhanced the compressive strength and modulus. In the case of 0.1% and 0.2% filled MWCNTs, the compressive strength was increased by 17–362%, 84–117%, and 39%, respectively, for UD, CP, and AP laminates. Similarly, the addition of 0.1–0.2% GNP, 86–499%, 104–158%, and 83–145% led to an increase in compressive strength observed for UD, CP, and AP laminates, respectively. However, the hybrid combination of 0.2% (MWCNT + GNP) showed superior improvement in compressive strength by 510%, 216%, and 205%, respectively, for UD, CP, and AP laminates. Beyond 0.2%, i.e., at 0.3% addition of the nanofillers, the properties were degraded due to agglomeration. Hence, the hybrid combination of 0.2% (MWCNT + GNP) significantly improved GE composites’ compressive properties. The summarised quasistatic compressive properties of GE and its nanocomposites with UD, CP, and AP configurations are given in Table 3.

**Figure 9:**
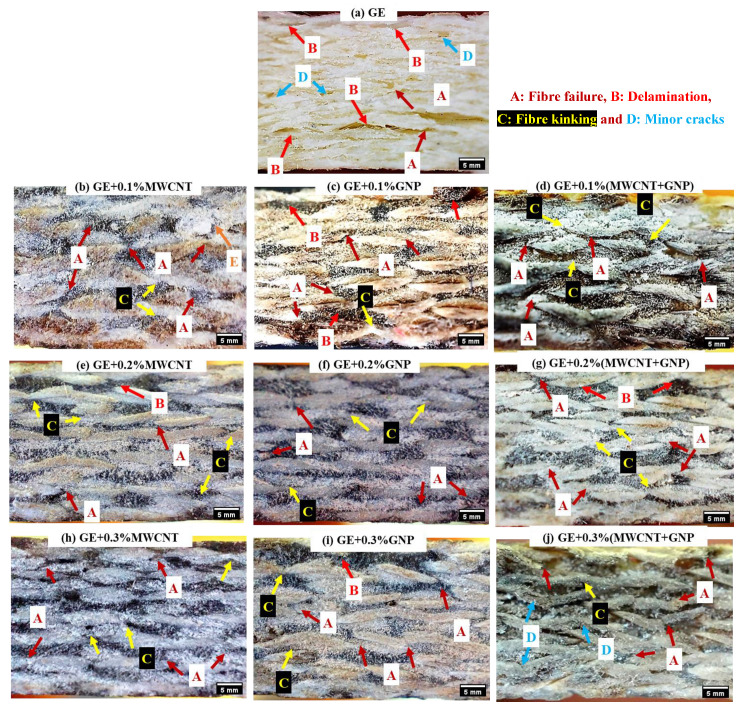
Compressive failure of GE and its nanocomposites (UD).

The compressive failure in tested samples is shown in Figure 9 at 20× magnification. Pure GE (Figure 9a) failed due to delamination and fibre failure. In the case of 0.1% of fillers, the delamination was reduced, but fibre failure was evident in all samples. As the percentage of fillers increased from 0.1 to 0.3, the reduction in failure was insignificant at the macroscopic level. Typically observed failure modes were matrix cracking, fibre kinking, delamination, and fibre breakage. The hybrid combination of MWCNT and GNP reduced the delamination compared to the virgin GE sample. In contrast, matrix cracking and delamination were mostly observed in the 0.3% MWCNT and GNP-filled individual neat glass epoxy composites. The dispersion process could not break the agglomeration of the composites with 0.3% MWCNT and GNP composites.

### 3.3. Flexural Properties

**Figure 10:**
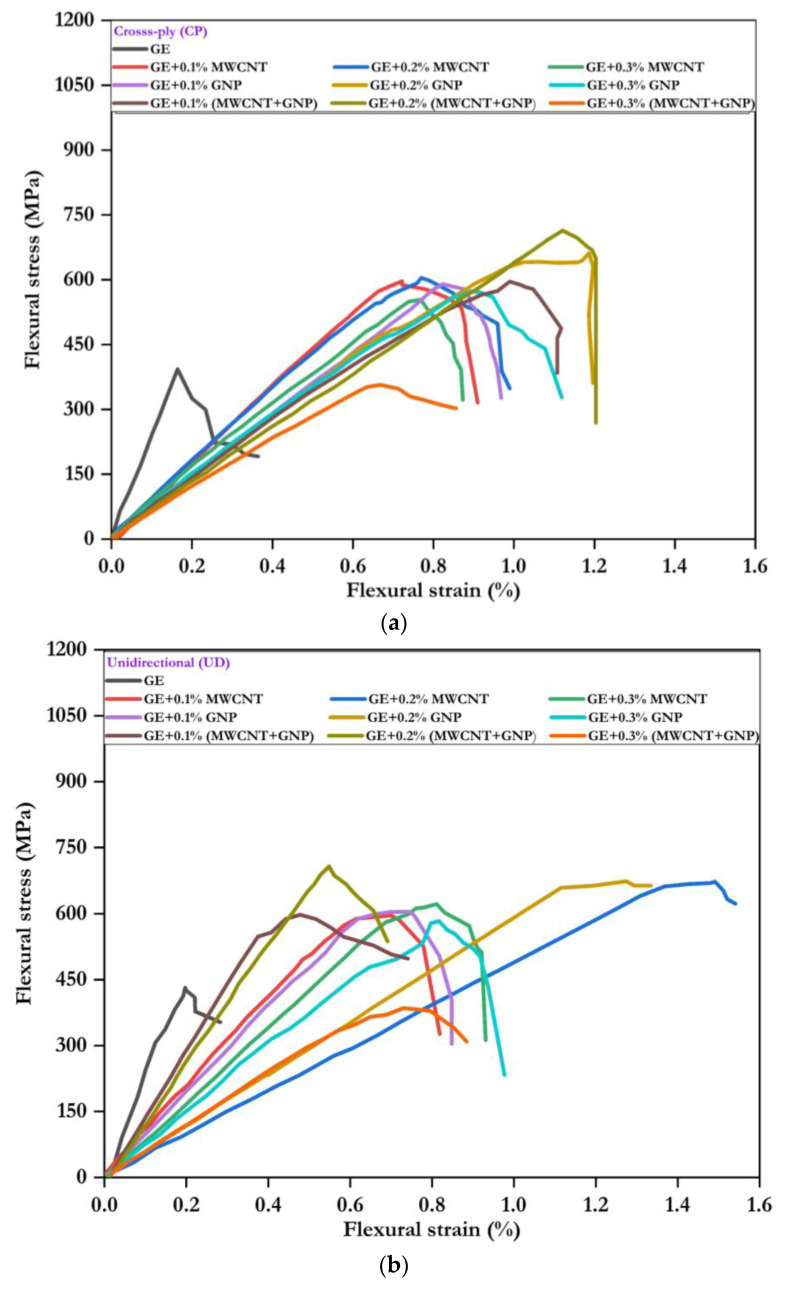

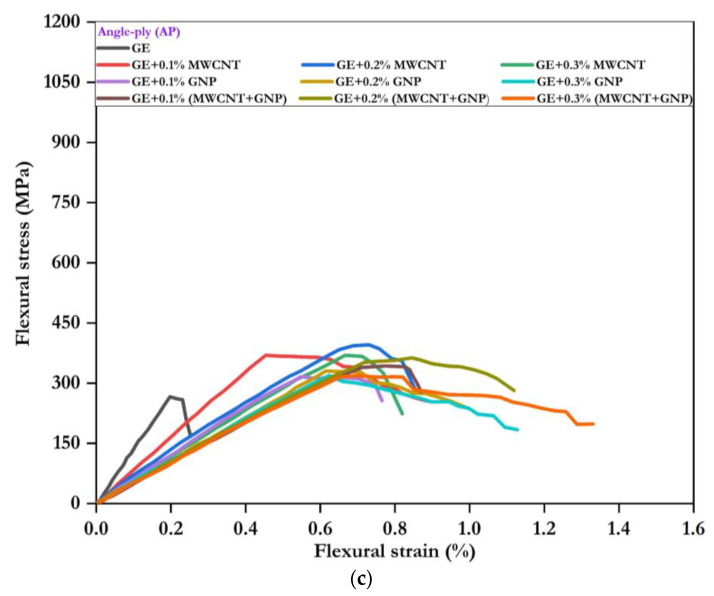
Flexural stress–strain response: (**a**) UD, (**b**) CP, and (**c**) AP.

The flexural stress–strain response for the neat UD, CP, and AP laminates with variable weight fractions of nanofillers is shown in Figure 10. Up to a strain of almost 0.2%, the response of all composites was similar, except for the difference in slopes. The flexural stress increased in all composites up to the first drop in the load, beyond which plastic deformation occurred. After the elastic limit, damage evolution varied according to the constituents of the laminates. In Figure 10a–c, it was observed that the UD laminate showed superior flexural performance over CP and AP samples. The response showed the peak stress of the specimen with substantial nonlinear deformation. The flexural performance response was improved from the neat laminate to laminates with nanofillers, irrespective of their stacking sequence.

**Figure 11:**
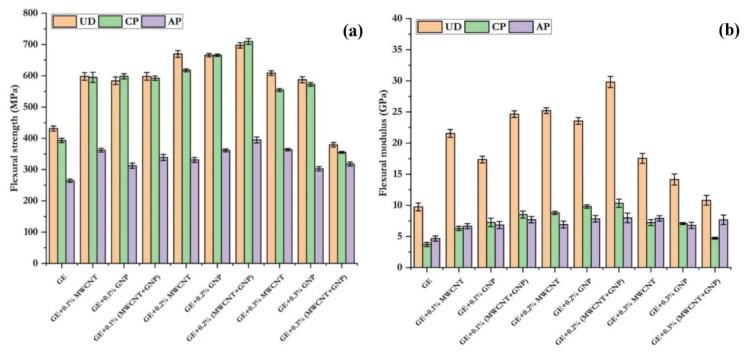
Flexural properties of UD, CP, and AP composites: (**a**) flexural strength and (**b**) flexural modulus.

The flexural response showed a similar trend in the individual and combined effect of MWCNT and GNP, which increased the flexural strength and flexural modulus in all GE composites, as shown in Figure 11. The flexural strength of UD, CP, and AP laminates was increased by 39–53%, 51–57%, and 25–37% with the addition of 0.1–0.2% MWCNTs. Additionally, adding 0.1–0.2% GNP increased the flexural strength by 35–54%, 52–69%, and 18–37% for UD, CP, and AP laminates, respectively. The hybrid combination of 0.1–0.2% (MWCNT + GNP) exhibited 39–62%, 51–80%, and 28–49% higher flexural strength for UD, CP, and AP laminates, respectively. The improvement in flexural properties of 0.2% (MWCNT + GNP) filled GE-CP loading composites could be attributed to the high mechanical properties and an improved fibre/matrix interface bonding due to the addition of 0.2% (MWCNT + GNP) to the epoxy. However, properties were increased only up to 0.2% (MWCNT + GNP), and a further increase in percentage showed degradation of properties. This may be due to the agglomeration of MWCNT + GNP in the epoxy matrix at higher concentrations, as confirmed by the SEM images. The neat GE composite, due to the addition of MWCNT, GNP, and MWCNT + GNP, had a more significant aspect ratio, leading to the prevention of crack initiation and dissemination in the epoxy matrix. The combination of 0.2% (MWCNT + GNP) displayed the highest flexural strength over the other combinations, as shown in Figure 11a. Table 4 shows the flexural properties of UD, CP, and AP laminates with different wt.% of nanofillers.

**Figure 12:**
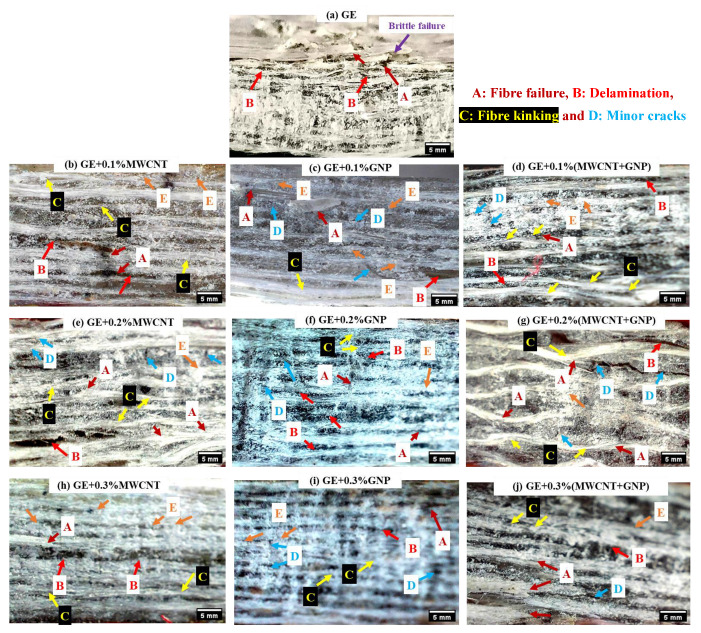
Flexural failure of GE and its nanocomposites (UD).

Figure 12 shows the flexural failure of samples through optical micrographs. Although the damage initiation occurred on the compression side (loading side), the failure on the tension side was less evident, as the specimen showed almost negligible deformation. In all the laminates, on the compression side (top surface), failure was due to fibre failure, delamination, matrix cracking, and fibre kinking. The reported failure modes were based on observing failure in the through thickness direction of the sample. There could be other failure modes that might have occurred earlier than those identified. The identified failure modes are highlighted in Figure 12.

**Figure 13:**
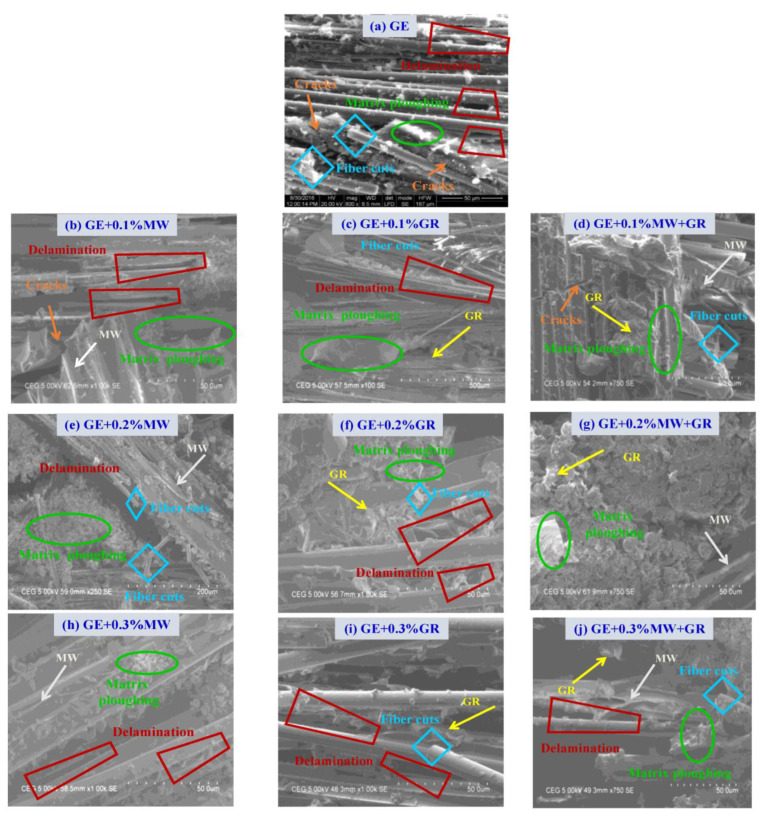
SEM micrographs of flexural failure in GE and its nanocomposites (UD).

The SEM micrographs of flexural failure for the UD samples with 0.1%, 0.2%, and 0.3% nanofillers are shown in Figure 13a–j. Pure GE (Figure 13a) showed fibre cuts with delamination, visible matrix cracks at multiple locations, and matrix ploughing. In the case of GE samples filled with 0.1% fillers (Figure 13b–d), delamination, matrix ploughing, and fibre cuts were typical failure modes. However, shear cracks were observed only in samples filled with MWCNT, i.e., in GE + MWCNT and GE + 0.1% (MWCNT + GNP). This might indicate that adding GNP particles prevent crack propagation under flexural loading. The micrographs of GE filled with 0.2% and 0.3% fillers (Figure 13e–g) showed excellent dispersion of nanofillers. Some of the MWCNT and GNP were broken in a brittle manner, and some were pulled out from the surface of the GE. Micrographs in Figure 13c,f,i showed an excellent bridging effect in the interfacial region of the glass fibre, nanofillers, and epoxy matrix. Due to the increase in the wt.% of nanofillers, crack propagation was prevented, showing improved performance. Finally, from these micrographs, a combined effect of 0.2% MWCNT + GNP nanofillers suggested with both resin and fibre suggested a better improvement in the flexural performance of the nanocomposites.

### 3.4. ILSS Properties

**Figure 14:**
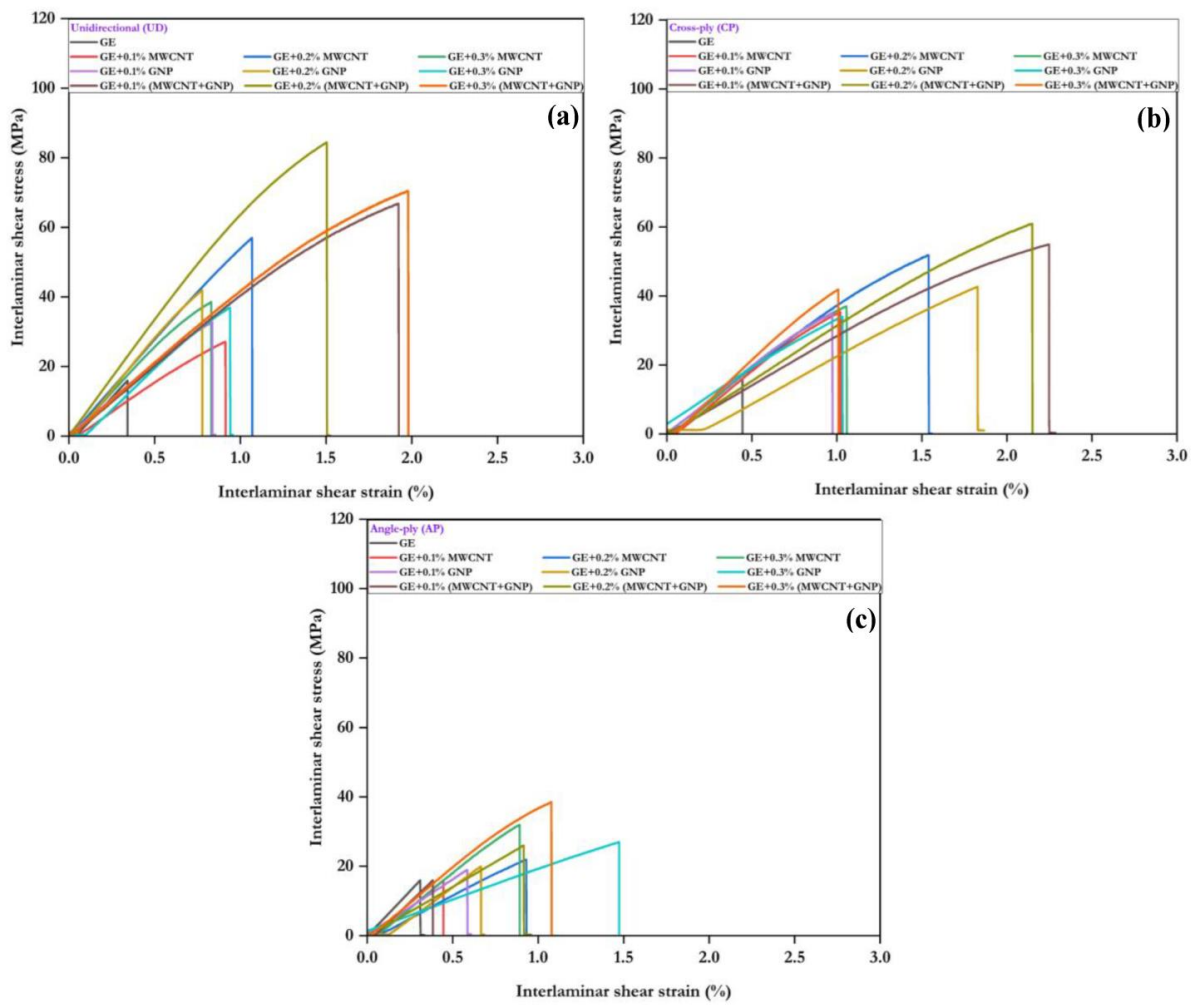
Interlaminar stress–strain response: (**a**) UD, (**b**) CP, and (**c**) AP.

The ILSS response of neat GE laminates and their nanocomposites is shown in Figure 14. In Figure 14, it was evident that the addition of MWCNT and GNP fillers improves the ILSS response of GE composites. However, the response was changed based on the constituents of the composites and their configuration. Among all the configurations, UD composites exhibited better performance than the CP and AP composites. In the case of pure GE composite, the response increased linearly and reached maximum stress with strain and then failed abruptly, indicating the brittle nature of the sample. When nanofillers were added, the stress increased linearly almost halfway and then exhibited nonlinearity, indicating plastic deformation. Beyond the nonlinearity, the response reached a peak load and dropped suddenly. The ILSS response increased up to a filler percentage of 0.2%; beyond this, it decreased. The ILSS response of CP configurations with nanofillers was better than the UD and AP composites.

**Figure 15:**
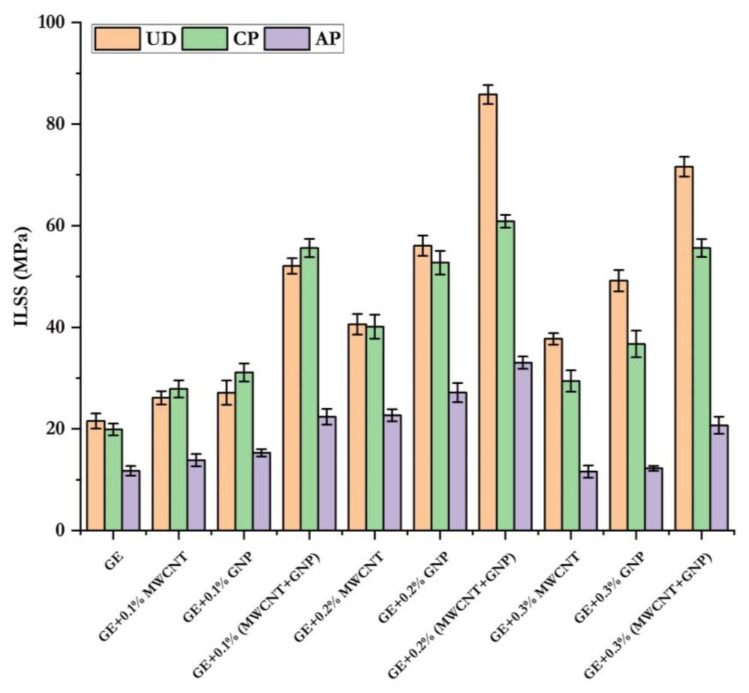
Comparison of ILSS for different laminates.

Figure 15 shows the ILSS values of GE composites with different nanofillers and configurations. The ILSS values were compared in this figure. Adding 0.1–0.2% of MWCNT and GNP separately improved the ILSS of UD, CP, and AP laminates by 21–160%, 41–165%, and 18–131%, respectively. The hybrid combination (MWCNT + GNP) greatly enhanced the ILSS of UD, CP, and AP by 142–298%, 179–206%, and 90–181%, respectively. The decrease in ILSS at 0.3% was due to the agglomeration of nanofillers. Table 5 shows the ILSS values of UD, CP, and AP laminates with different wt.% of nanofillers.

**Figure 16:**
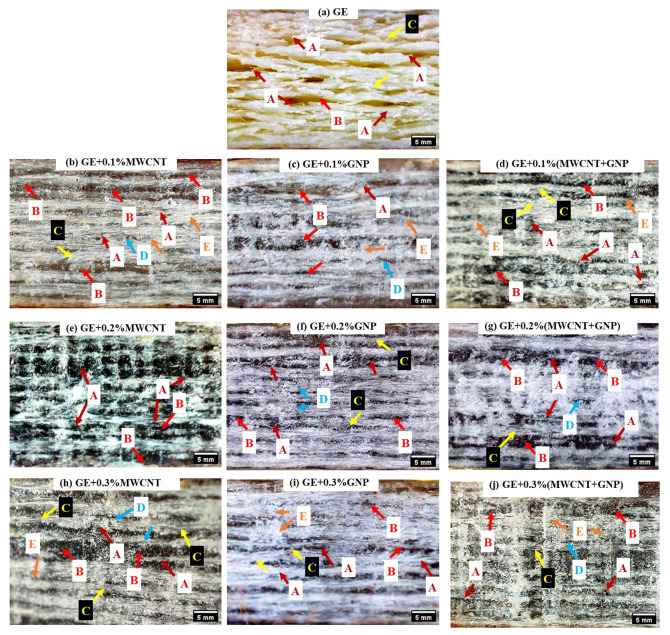
ILSS failure of GE and its nanocomposites (UD).

The interlaminar shear failure of neat and nanocomposites are shown in Figure 16, using optical micrographs. The GE composite (Figure 16a) clearly showed fibre failure and delamination. However, in 0.1 and 0.2% GE nanocomposites (Figure 16b–g), there was no evidence of delamination, which justifies the increased ILSS value. In the case of GE nanocomposites with 0.1–0.2% of MWCNT and GNP nanofillers, minor delamination with fibre failure was evident. However, the failure modes were insignificant in 0.1–0.2% (MWCNT + GNP) laminates.

**Figure 17:**
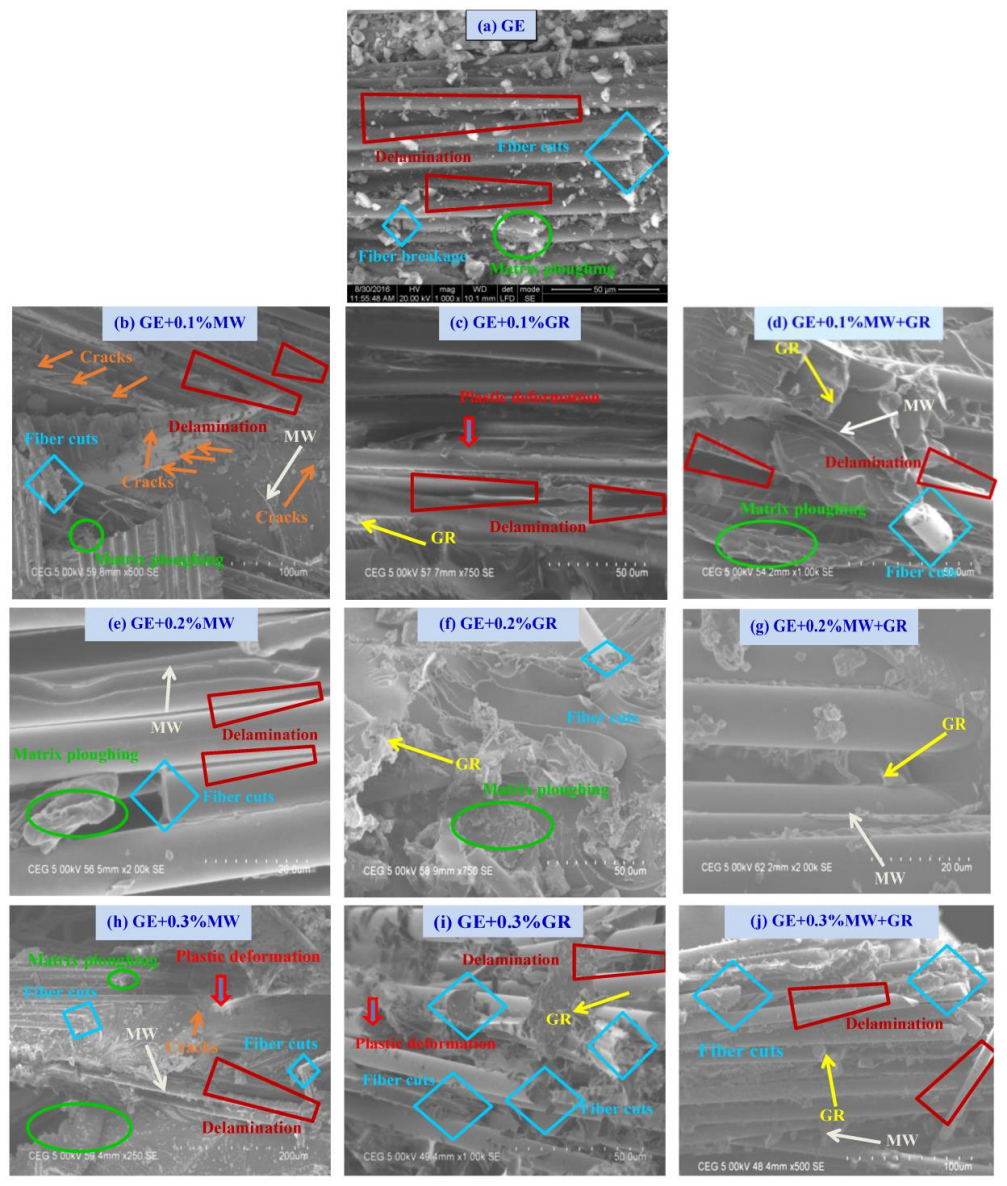
SEM micrographs of interlaminar shear failure in GE and its nanocomposites (UD).

The interlaminar shear failure of GE and its nanocomposites is shown in Figure 17 through SEM micrographs. In the neat GE sample (Figure 17a), the matrix fracture surface was even, and a small number of cracks were identified at the fibre matrix interface. The poor interface between the fibre and matrix was due to the void’s presence and the resin’s non-uniform distribution. In the case of samples with 0.1% nanofillers (Figure 17b–d), fibre cuts, delamination, and matrix ploughing were observed. In addition, samples with MWCNT fillers (Figure 17b) exhibited cracks in different directions at multiple locations. As the filler percentage increased (Figure 17e–j), the failure in the samples was reduced due to better interfacial bonding, which improved the ILSS values. The uniformly distributed nanofillers were observed but randomly placed in the matrix. The bonding between fibre surfaces with resin significantly improved plastic deformation due to the addition of nanofillers, which subsequently enhanced the mechanical properties. These nanofillers strengthen the elements that act as reinforcement and increase load carrying capacity of the composite. The sample with a hybrid combination of MWCNT + GNP with glass exhibited a bridging effect between fibre and resin, which enhanced the ILSS of the composite structures.

## 4. Conclusions

Nanofillers have recently attracted attraction as additives to composite laminates to enhance their mechanical properties. In the current investigation, MWCNTs and GNP nanofillers were used independently and as a hybrid combination at different weight fractions (0.1–0.3%). The laminates were prepared with different configurations and tests were conducted per ASTM standards. Based on the outcomes, the following conclusions were made:

- The addition of MWCNT, GNP, and a hybrid combination of these enhanced the compressive strength by 17–499%, 84–158%, and 39–145%, respectively, for UD, CP, and AP laminates. The configuration hierarchy for compressive strength was UD > CP > AP for all nanofill combinations.
- The addition of MWCNT and GNP increased the flexural strength of UD, CP, and AP laminates by 39–54%, 51–69%, and 37%, respectively. The hybrid combination of 0.1–0.2% (MWCNT + GNP) exhibited superior flexural strength by 39–62%, 51–80%, and 28–49% higher flexural strength for UD, CP, and AP laminates, respectively.
- MWCNT and GNP combined enhanced the ILSS of UD, CP, and AP laminates 142–298%, 179–206%, and 90–181%, respectively.
- The weight fraction of the nanoadditive played a significant role in the performance enhancement, and a 0.2% weight fraction was found to be optimal for strong bonding between the fibres.
- The combination of MWCNT + GNP yielded better performance over the independent constituents at a 0.2% weight fraction. It found better interfacial bonding due to the combined birding effect.
- Morphological (SEM and optical microscopy) studies revealed the bridging effect of MWCNT. GNP promoted good adhesion between the glass fibre and matrix by modifying the matrix adhesive properties, and hence properties of the composite increased.

## Figures and Tables

**Table 1:** Nano additives and their influence on improving the mechanical properties.

Ref. No.	CNF/Nano Additives					Strength Properties				
	AMNC	MWCNT	CNF	NC	SiC	Tensile	Compressive	Flexural	Shear	Fatigue
[16]		✓				✓				
[20]		✓				✓				✓
[23]				✓						✓
[25]		✓				✓		✓		
[26]		✓				✓		✓	✓	
[27]		✓				✓				
[30]		✓				✓				
[31]		✓					✓			
[32]	✓							✓	✓	
[33]			✓						✓	
[34]					✓	✓				
[36]		✓				✓				
[38]			✓					✓		
[44]			✓				✓	✓		
[45]					✓		✓			
[50]			✓			✓		✓		✓
[51]			✓				✓		✓	
[52]				✓				✓		

**Table 2:** Physical and mechanical properties of nanofillers and LY-556 epoxy.

Type of Material	Property	Value
Graphene (GNP)	Purity	>99%
	Thickness	5–10 nm
	Length	5–10 microns
	Surface Area	190 m^2^/g
	Thermal Conductivity	3000 w/m-K
	Tensile Strength	~1100 GPa
	Tensile Modulus	>1000 GPa
	Electrical Conductivity	10^7^ Siemens/m
Multiwalled Carbon nanotubes (MWCNTs)	Diameter	12–15 nm
	Length	3–10 microns
	Purity	>97%
	Surface Area	250–270 m^2^/g
	Tensile Strength	30~180 GPa
	Thermal Conductivity	6000 w/m-k
	Electrical Conductivity	6000 S/cm
	Modulus	1~2 TPa
LY-556 Epoxy matrix	Viscosity at 25 °C	10,000–12,000 mPa s
	Density at 25 °C	1.15–1.20 g/cm^3^

**Table 3:** Compressive properties of UD, CP, and AP nanocomposites.

Composite	Compressive Strength (MPa)			Compressive Modulus (GPa)		
	UD	CP	AP	UD	CP	AP
GE	14.65 ± 5.12	12.45 ± 4.02	4.64 ± 2.13	10.27 ± 0.41	9.16 ± 0.71	4.65 ± 0.23
GE + 0.1% MWCNT	17.21 ± 2.47	22.89 ± 1.54	2.33 ± 0.23	7.37 ± 2.31	10.73 ± 0.22	1.15 ± 0.82
GE + 0.1% GNP	27.24 ± 9.33	25.45 ± 1.51	8.49 ± 2.43	12.46 ± 1.43	12.61 ± 3.11	2.38 ± 1.61
GE + 0.1% (MWCNT + GNP)	85.30 ± 18.21	30.07 ± 3.53	8.68 ± 6.21	14.51 ± 1.48	23.21 ± 2.71	2.65 ± 1.82
GE + 0.2% MWCNT	67.71 ± 5.21	27.07 ± 2.61	6.46 ± 2.72	13.14 ± 1.41	9.46 ± 2.43	0.94 ± 0.21
GE + 0.2%GNP	87.80 ± 0.55	32.11 ± 2.41	11.35 ± 2.82	14.27 ± 4.22	20.91 ± 3.42	1.75 ± 0.28
GE + 0.2% (MWCNT + GNP)	89.35 ± 18.21	39.34 ± 5.11	14.16 ± 2.61	18.33 ± 3.6	22.09 ± 5.12	5.53 ± 3.11
GE + 0.3% MWCNT	24.36 ± 2.62	21.34 ± 3.14	5.85 ± 2.43	8.11 ± 0.79	6.61 ± 1.22	1.32 ± 0.42
GE + 0.3% GNP	67.78 ± 37.6	26.01 ± 4.42	9.48 ± 3.63	12.52 ± 1.72	18.91 ± 2.84	2.53 ± 0.91
GE + 0.3% (MWCNT + GNP)	80.91 ± 21.32	30.62 ± 4.22	10.16 ± 2.62	12.8 ± 6.11	23.16 ± 3.12	2.85 ± 0.16

**Table 4:** Flexural properties of neat and nanofillers reinforced UD, CP, and AP nanocomposites.

Composites	UD		CP		AP	
	Flexural Strength (MPa)	Flexural Modulus (GPa)	Flexural Strength (MPa)	Flexural Modulus (GPa)	Flexural Strength (MPa)	Flexural Modulus (GPa)
GE	431.27 ± 8.10	9.75 ± 0.81	393.25 ± 6.37	3.70 ± 0.72	264.53 ± 5.36	4.66 ± 1.41
GE + 0.1% MWCNT	597.92 ± 9.40	21.54 ± 1.14	594.83 ± 15.30	6.29 ± 0.54	361.92 ± 6.02	6.64 ± 2.22
GE + 0.1% GNP	583.78 ± 9.90	17.36 ± 0.11	598.09 ± 8.25	7.25 ± 1.22	312.43 ± 8.40	6.83 ± 1.84
GE + 0.1% (MWCNT + GNP)	597.96 ± 9.10	24.64 ± 2.50	592.41 ± 7.21	8.50 ± 2.81	338.73 ± 9.61	7.70 ± 1.02
GE + 0.2% MWCNT	669.60 ± 7.10	25.21 ± 0.12	617.36 ± 4.77	8.77 ± 2.44	330.93 ± 7.84	6.90 ± 1.31
GE + 0.2% GNP	666.13 ± 9.20	23.55 ± 0. 5	665.49 ± 3.64	9.80 ± 3.52	361.39 ± 4.65	7.82 ± 2.41
GE + 0.2% (MWCNT + GNP)	697.20 ± 9.10	29.81 ± 0.44	709.67 ± 9.34	10.32 ± 3.92	394.93 ± 9.58	7.98 ± 1.94
GE + 0.3% MWCNT	608.55 ± 9.80	17.55 ± 1.82	554.30 ± 5.08	7.23± 1.62	363.50 ± 3.72	7.89 ± 1.52
GE + 0.3% GNP	587.16 ± 5.10	14.13 ± 0.41	572.48 ± 5.75	7.05± 1.43	302.49 ± 6.80	6.77 ± 1.42
GE + 0.3% (MWCNT + GNP)	379.25 ± 8.10	10.80 ± 0.44	354.99 ± 3.04	4.70± 1.31	317.73 ± 6.47	7.66 ± 1.51

**Table 5:** ILSS of GE and its nanocomposites.

	ILSS (MPa)		
Composites	UD	CP	AP
GE	21.56 ± 1.12	19.90 ± 1.18	11.77 ± 0.94
GE + 0.1% MWCNT	26.13 ± 1.32	27.88 ± 1.68	13.85 ± 1.20
GE + 0.1% GNP	27.13 ± 2.12	31.12 ± 1.76	15.30 ± 0.70
GE + 0.1% (MWCNT + GNP)	52.09 ± 1.13	55.61 ± 1.80	22.40 ± 1.55
GE + 0.2% MWCNT	40.60 ± 2.03	40.13 ± 2.35	22.69 ± 1.19
GE + 0.2% GNP	56.07 ± 2.00	52.72 ± 2.32	27.16 ± 1.89
GE + 0.2% (MWCNT + GNP)	85.83 ± 1.86	60.87 ± 1.26	33.04 ± 1.20
GE + 0.3% MWCNT	37.74 ± 1.13	29.45 ± 2.09	11.62 ± 1.22
GE + 0.3% GNP	49.18 ± 2.10	36.73 ± 2.62	12.5 ± 0.48
GE + 0.3% (MWCNT + GNP)	71.61 ± 1.93	55.61 ± 1.75	20.72 ± 1.68

## Data Availability

The data presented in this study are available on request from the corresponding author.

## Supplementary Materials

The following supporting information can be downloaded at: https://www.mdpi.com/article/10.3390/polym15051189/s1. The supplementary file presents the flexural and interlaminar shear failure of CP and AP laminates through SEM images. Figure S1: Flexural failure of GE and its nanocomposites (CP). Figure S2: Flexural failure of GE and its nanocomposites (AP). Figure S3: Interlaminar shear failure of GE and its nanocomposites (CP). Figure S4: Interlaminar shear failure of GE and its nanocomposites (AP).
