# Supplementary material for: Enhancement in Mechanical Properties of Glass/Epoxy Composites by a Hybrid Combination of Multi-Walled Carbon Nanotubes and Graphene Nanoparticles
Source: Polymers (Basel). 2023 Feb 27;15(5):1189. doi: 10.3390/polym15051189 (PMC10007175; doi:10.3390/polym15051189)
Supplement: Supplementary file 1 [file polymers-15-01189-s001.zip › polymers-2194628-supplementary.pdf]

__**Flexural failure**__

__**Cross-ply**__

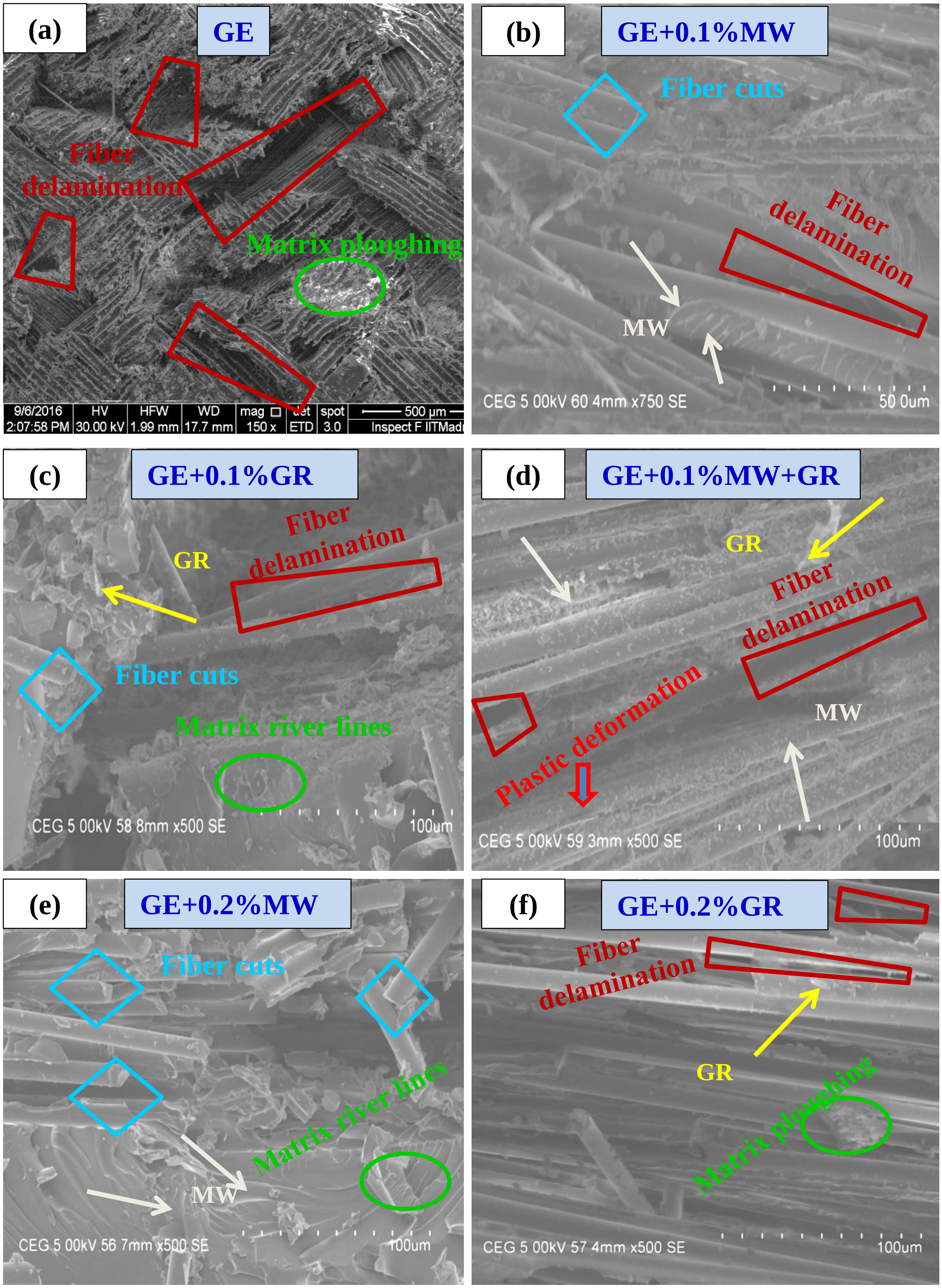

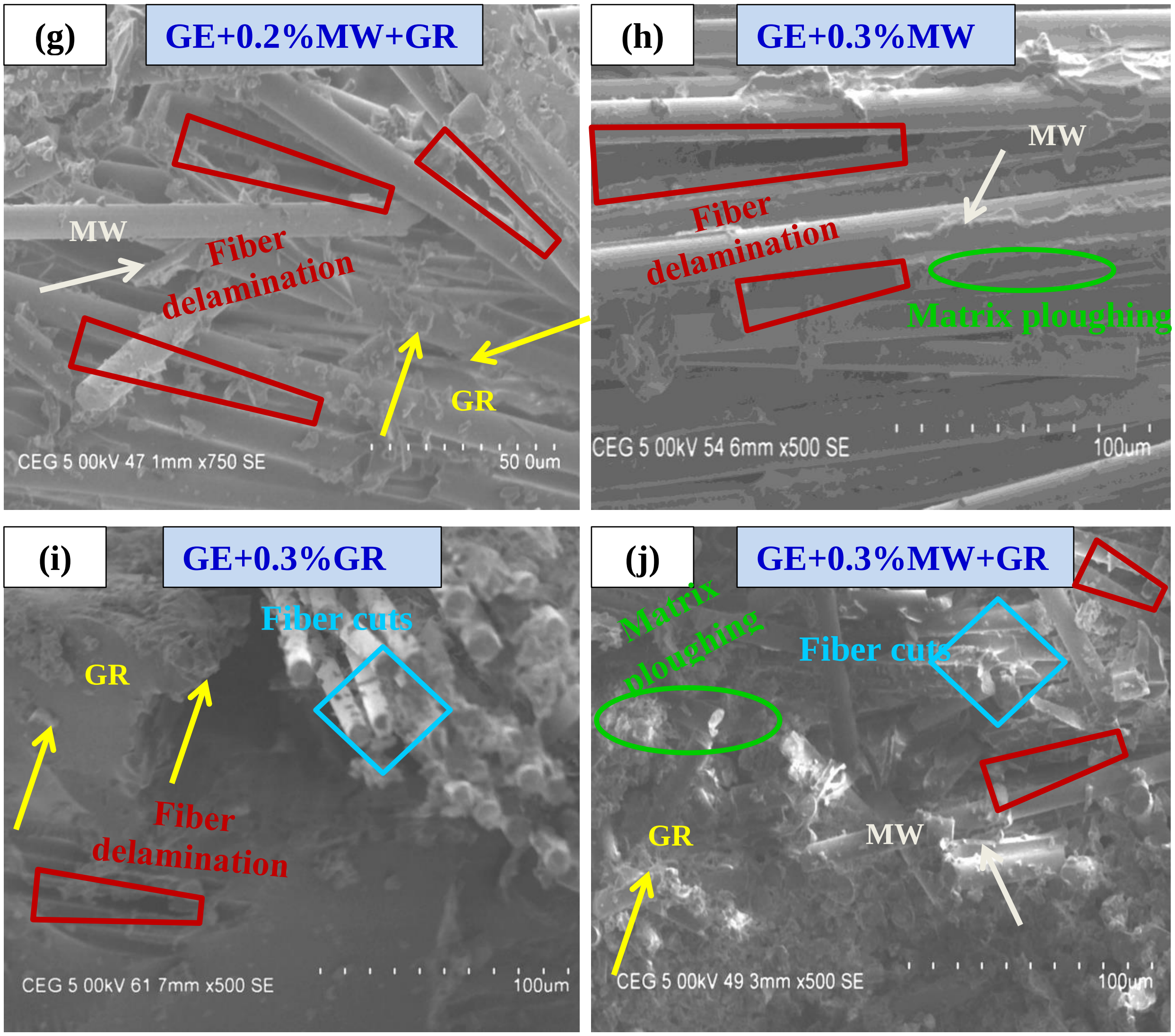

Figure S1. Flexural failure of GE and its nanocomposites (CP).

Angle-ply

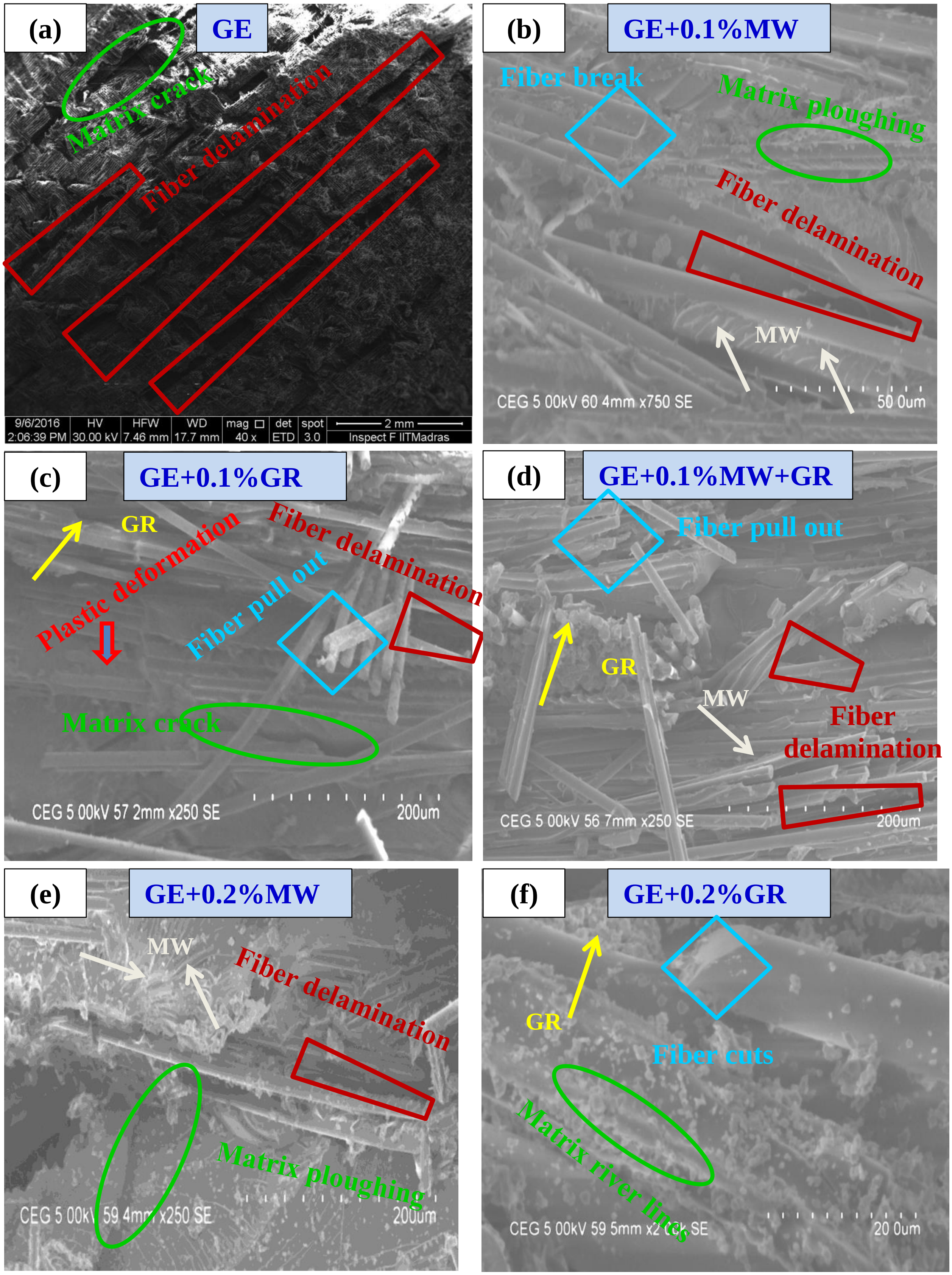

(a)
GE
Matrix crack
Fiber delamination
9/6/2016 2:06:39 PM
HV 30.00 kV
HFW 7.46 mm
WD 17.7 mm
mag 40 x
det ETD
spot 3.0
2 mm
Inspect F IITMadras
(b)
GE+0.1%MW
Fiber break
Matrix ploughing
Fiber delamination
MW
CEG 5 00kV 60 4mm x750 SE
50 0um
(c)
GE+0.1%GR
GR
Plastic deformation
Fiber delamination
Fiber pull out
Matrix crack
CEG 5 00kV 57 2mm x250 SE
200um
(d)
GE+0.1%MW+GR
Fiber pull out
GR
MW
Fiber delamination
CEG 5 00kV 56 7mm x250 SE
200um
(e)
GE+0.2%MW
MW
Fiber delamination
Matrix ploughing
200um
(f)
GE+0.2%GR
GR
Fiber cuts
Matrix river lines
20 0um

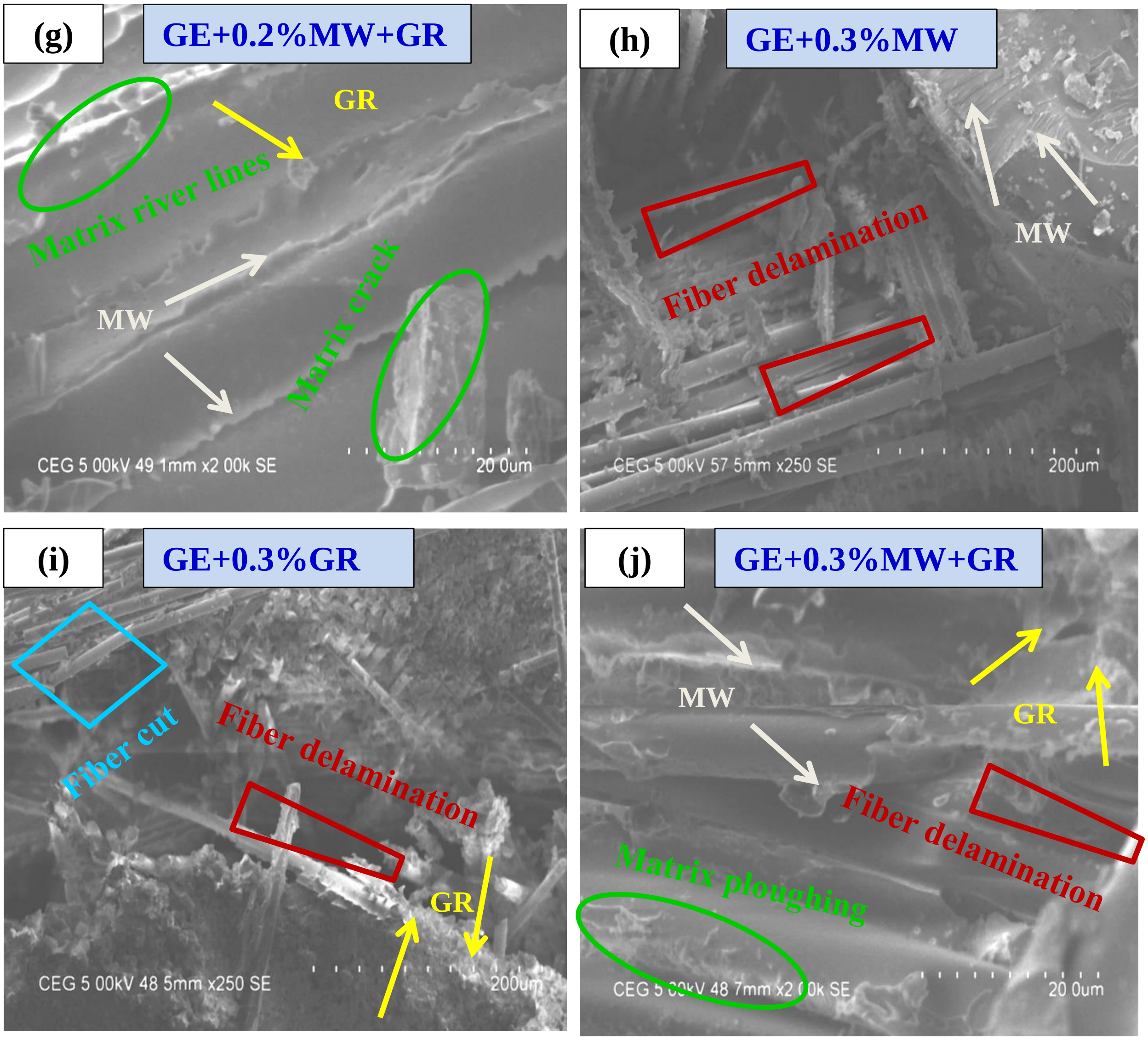

Figure S2. Flexural failure of GE and its nanocomposites (AP).

ILSS Failure

Cross-ply

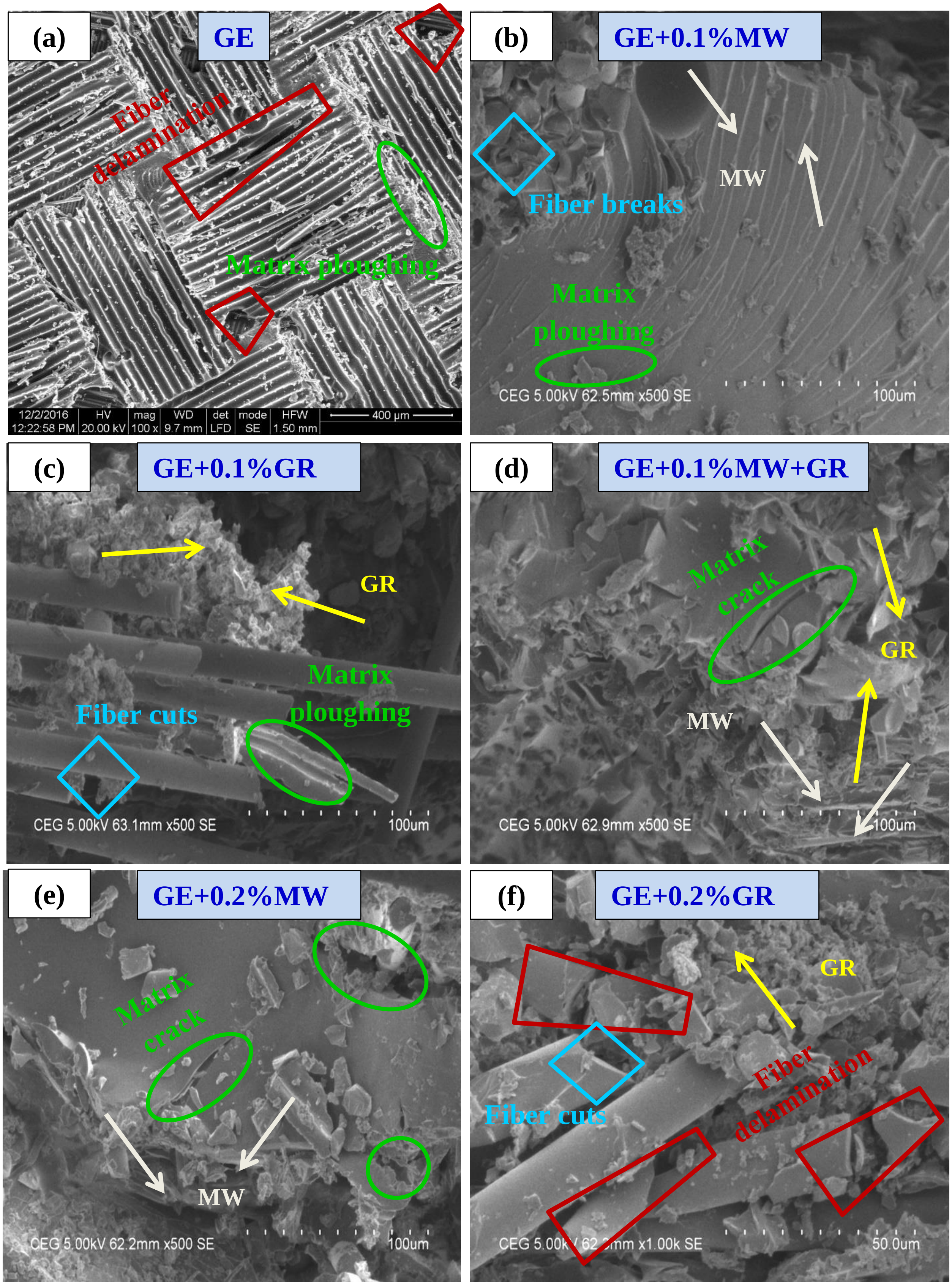

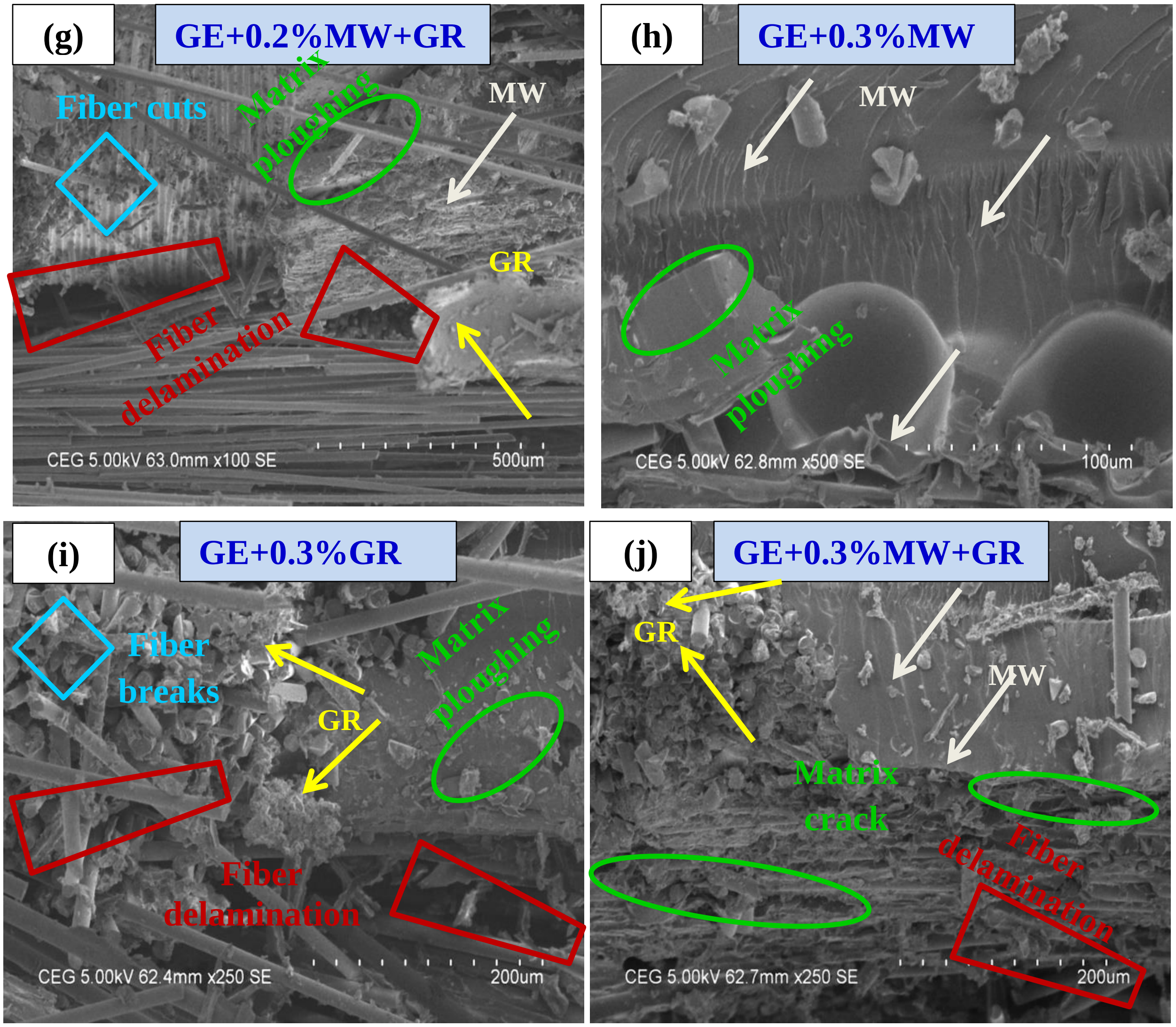

Figure S3. Interlaminar shear failure of GE and its nanocomposites (CP).

Angle-ply

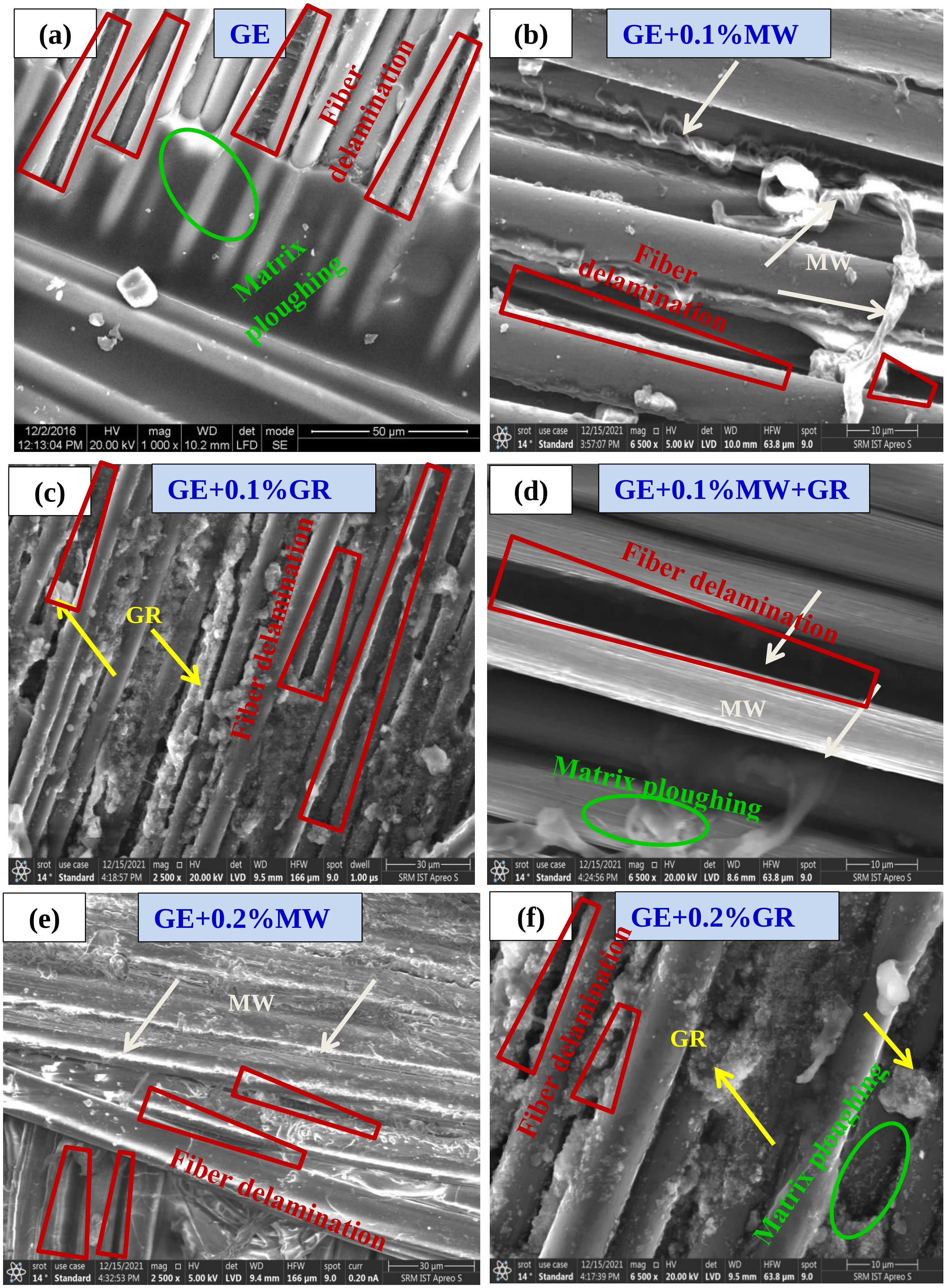

(a)
GE
Fiber delamination
Matrix ploughing
(b)
GE+0.1%MW
MW
Fiber delamination
(c)
GE+0.1%GR
GR
Fiber delamination
(d)
GE+0.1%MW+GR
Fiber delamination
MW
Matrix ploughing
(e)
GE+0.2%MW
MW
Fiber delamination
(f)
GE+0.2%GR
Fiber delamination
GR
Matrix ploughing

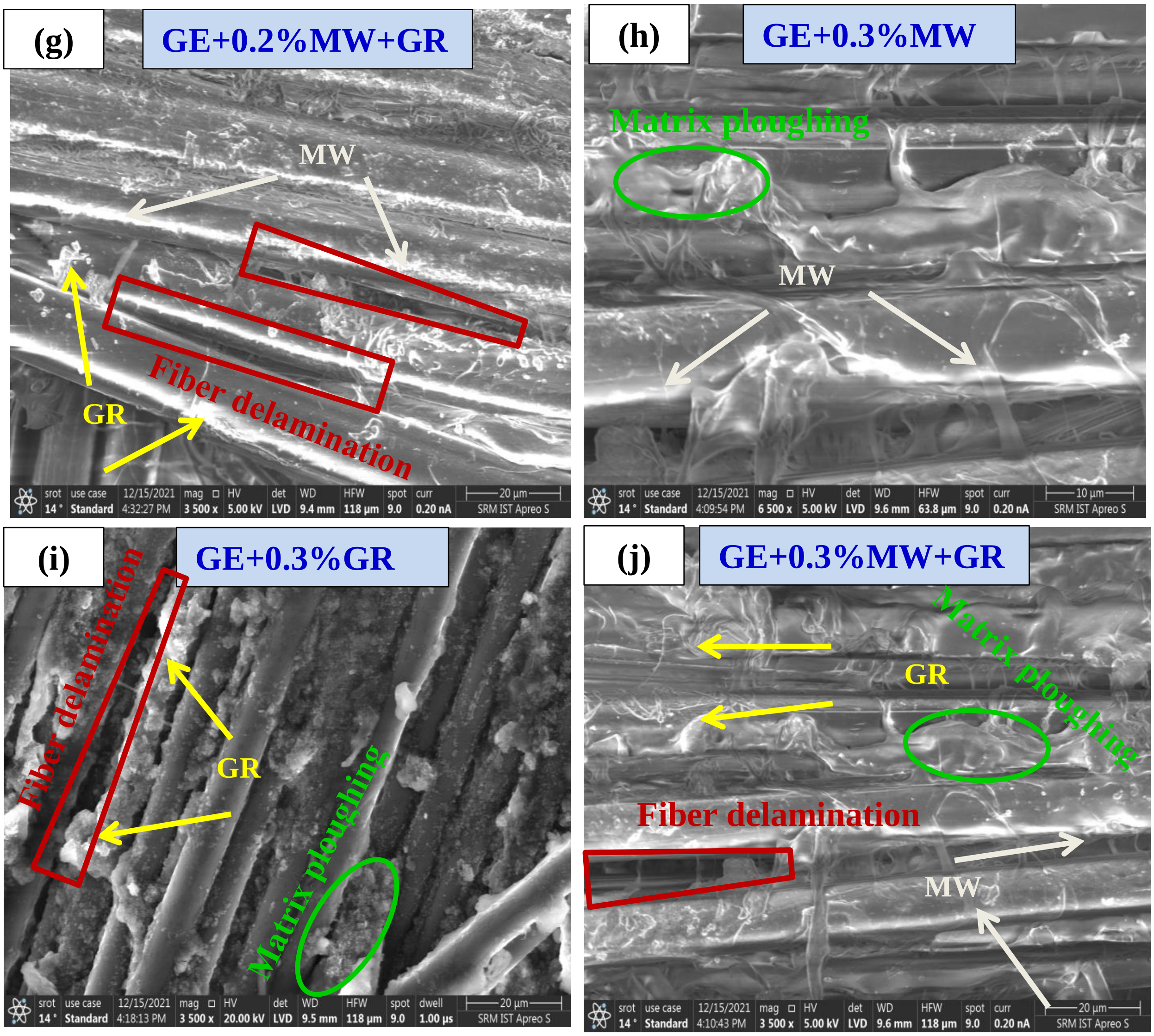

Figure S4. Interlaminar shear failure of GE and its nanocomposites (AP).
